# Selection and demography drive range-wide patterns of MHC-DRB variation in mule deer

**DOI:** 10.1186/s12862-022-01998-8

**Published:** 2022-04-06

**Authors:** Rachel M. Cook, Brittany Suttner, Rachael M. Giglio, Margaret L. Haines, Emily K. Latch

**Affiliations:** 1 Department of Biological Sciences, University of Wisconsin-Milwaukee, Milwaukee, WI 53211 USA; 2 GenoTwin, Bridgewater, MA USA; 3 School of Biological Sciences, Monash University, Clayton, VIC Australia; 4 USDA, Wildlife Services, National Wildlife Research Center, Wildlife Genetics Lab, Fort Collins, CO USA

**Keywords:** Major histocompatibility complex, Natural selection, Adaptation, Genetic variation, Parasite-mediated selection, Genetic drift, Historical demography, Microsatellites, Next-generation sequencing, Cervidae

## Abstract

**Background:**

Standing genetic variation is important especially in immune response-related genes because of threats to wild populations like the emergence of novel pathogens. Genetic variation at the major histocompatibility complex (MHC), which is crucial in activating the adaptive immune response, is influenced by both natural selection and historical population demography, and their relative roles can be difficult to disentangle. To provide insight into the influences of natural selection and demography on MHC evolution in large populations, we analyzed geographic patterns of variation at the MHC class II DRB exon 2 locus in mule deer (*Odocoileus hemionus*) using sequence data collected across their entire broad range.

**Results:**

We identified 31 new MHC-DRB alleles which were phylogenetically similar to other cervid MHC alleles, and one allele that was shared with white-tailed deer (*Odocoileus virginianus*). We found evidence for selection on the MHC including high dN/dS ratios, positive neutrality tests, deviations from Hardy–Weinberg Equilibrium (HWE) and a stronger pattern of isolation-by-distance (IBD) than expected under neutrality. Historical demography also shaped variation at the MHC, as indicated by similar spatial patterns of variation between MHC and microsatellite loci and a lack of association between genetic variation at either locus type and environmental variables.

**Conclusions:**

Our results show that both natural selection and historical demography are important drivers in the evolution of the MHC in mule deer and work together to shape functional variation and the evolution of the adaptive immune response in large, well-connected populations.

**Supplementary Information:**

The online version contains supplementary material available at 10.1186/s12862-022-01998-8.

## Background

The amount of genetic variation in a population is directly associated with its potential to adapt or evolve in new environments [1–3]. Populations capable of rapid adaptive responses can maintain biodiversity in rapidly changing environments, whereas those with lower adaptive capacity are vulnerable to population declines or extinction. Variation at functional genes involved in immune response is of increasing concern as wildlife diseases emerge as serious threats to populations, as seen in examples like amphibian chytridiomycosis [4, 5], white-nose syndrome in bats [6–9], and chronic wasting disease in ungulates [10–13]. Genes in the major histocompatibility complex (MHC) are some of the most polymorphic in vertebrate genomes, maintained by natural selection in response to an ever-changing environment of pathogens [14–18]. Specifically, exon 2 of the MHC DRB gene codes for extracellular antigen receptors and affects the activation of the adaptive immune system. Genetic diversity at this locus has evolved under pathogen-mediated selection in addition to demographic processes like genetic drift and gene flow that affect variation genome-wide, making the MHC an important region for understanding how functional diversity is maintained in natural populations [19–21].

Pathogen-mediated selection at the MHC can occur by way of directional selection in response to the presence of specific pathogens [22] or by balancing selection through non-mutually exclusive mechanisms including heterozygote advantage, negative frequency dependence, and fluctuating selection from heterogenous host–pathogen dynamics [23–27]. MHC variation in host populations is maintained by co-evolution between pathogen populations and the host immune response [28–32]. Sexual selection may also play a role as MHC allelic richness has been linked with sexually selected traits [18, 33–39]. Additionally, environmental factors may indirectly affect selection on the MHC by changing the pathogen load in an area [40, 41].

Historical population demography also influences MHC variation and can be challenging to disentangle from the effects of natural selection. Strong genetic drift in small and isolated populations reduces variation at all genes, including those of the MHC [42–47]. Comparing patterns of genetic variation at functional and neutral loci can help isolate the role of selection in maintaining MHC variation in natural populations. When the MHC evolves by neutral processes such as genetic drift and gene flow, the MHC would be expected to show patterns of variation similar to neutral loci [48–50], whereas selection would cause patterns of MHC variation to diverge from neutral loci, potentially mirroring patterns of variation in the selective agent [51–53]. For example, local adaptation is present where MHC differentiation is greater than that in neutral markers [51–53], balancing selection is present when MHC differentiation is weaker than at neutral markers [29, 31, 32], and MHC differentiation could be no different than that of neutral markers where weak selection or strong drift effects occur [49, 50]. Previous studies have used this comparative approach to learn more about adaptation at the MHC [42, 45–47, 54, 55]. However, many of these studies have considered populations that are small, isolated, or have experienced recent bottlenecks [32, 42, 45, 47, 49, 56–59]. We might expect different outcomes for large, highly connected populations, where genetic drift is not as prominent, and natural selection can guide adaptation of complex traits [60].

To better understand the relative roles of natural selection and demography on MHC evolution in large populations, we analyzed geographic patterns of variation at the MHC class II DRB exon 2 locus in mule deer (*Odocoileus hemionus*) and compared it to paired data from neutral loci based on microsatellite markers. Mule deer are distributed in diverse environments across a broad geographical range, spanning over 40° of latitude. In these varied environments, mule deer encounter a wide variety of infectious (viral, bacterial, prion) and parasitic (protozoan, helminths, arthropods) agents (Davidson et al. [61], Samuel et al. [62], Williams and Barker [63]). These agents can drive the evolution of ungulate MHC through natural selection [28, 64]. Historical demography will also shape variation at the MHC and genome-wide, with its role determined in large part by the species’ biogeographic history and long-term effective population size. Unlike previous studies, we take a broad look at mule deer MHC diversity by surveying across the entire geographic range. Mule deer populations are generally large, well connected, and genetically diverse [65], both today and historically. We have sampled core populations from a single evolutionary lineage, whose shared biogeographic history facilitates direct comparison of functional and neutral diversity range-wide. Comparing range-wide patterns of adaptive and neutral variation will help disentangle the relative influence of evolutionary mechanisms impacting the maintenance of MHC variation at a broad scale in mule deer.

## Results

### MHC sequencing and phylogeny

We successfully sequenced the entire 250 bp MHC class II DRB exon 2 in 384 individuals across 16 populations of mule deer. From sequencing, we obtained a total of 552,769 reads. Filtering resulted in an average of 1452 reads per individual (which ranged from 104 to 5000 reads) for 380 individuals (Additional file 1: Fig. S1 and Table S1). Four individuals, each from a different population, yielded few reads (< 100) and were removed from further analysis (n for each population shown in Tables 1 and 2). Following the characteristic high polymorphism rates of MHC genes, we found 69 variable sites, corresponding to amino acid changes at 37 of the 83 codons (Additional file 1: Fig. S2). All alleles were unique at the amino acid level. The number of amino acid differences between alleles ranged from 1 to 26. Each individual had a maximum of two alleles, consistent with the presence of a single MHC-DRB locus (Additional file 1: Fig. S1). One individual showed three alleles, but one of the called alleles was an artifact with low read count (Population AB-728, read numbers included 553, 514 and 179) (Additional file 1: Fig. S1, Table S1). Zero alleles were called for six individuals, however, read numbers permitted unambiguous manual allele assignment (Additional file 1: Fig. S1).We observed 31 new alleles and one allele (Odvi-DRB*09) previously recorded in white-tailed deer [66]. The new mule deer alleles (Odhe-DRB*01-31) were deposited in GenBank under accession numbers MZ450871-MZ450901. Although four alleles (Odhe-DRB*23, Odhe-DRB*25, Odhe-DRB*27, and Odhe-DRB*31) were observed only once, each had over 500 reads when present and thus were considered to be true alleles and included in downstream analyses (Table 1, Additional file 1: Table S1, Fig. S1).

**Table 1:** Summary of MHC class II DRB2 variation by population

Population	n	MHC								
		A	A_P_	H_O_	H_E_	HWE p value	Tajima’s D	Fu’s Fs	Fu and Li’s D*	Fu and Li’s F*
AB-728	24	13	0	0.667	0.837	*	1.25	11.69	1.31	1.54
AB-OR	23	14	0	0.696	0.868	**	1.48	10.38	1.07	1.46
AZ-30	24	10	0	0.750	0.867	ns	1.32	17.42	1.57*	1.76*
AZ-KF	24	11	0	0.750	0.814	ns	1.16	13.65	1.12	1.36
CO-SJ	24	10	0	0.583	0.839	*	1.65	18.13	1.67**	1.99**
CO-ST	24	13	0	0.583	0.859	ns	0.82	10.96	1.11	1.20
KS-BD	24	11	0	0.625	0.859	*	0.67	12.46	2.04**	1.84*
MT-RV	24	16	3	0.708	0.893	**	1.42	6.95	0.96	1.35
ND-SW	24	15	0	0.583	0.832	***	0.61	7.36	1.21	1.18
NV-PC	24	17	0	0.750	0.917	ns	0.91	6.46	1.60*	1.61
SD-CU	24	10	0	0.542	0.824	***	1.06	15.58	0.77	1.05
SK-14	23	11	0	0.435	0.831	***	0.98	12.64	1.76**	1.76*
SO-CS	24	8	0	0.583	0.829	ns	1.66	20.09	1.04	1.51
TX-AL	23	11	2	0.783	0.836	ns	1.42	15.52	1.10	1.45
WY-SH	24	17	0	0.833	0.880	ns	1.06	6.66	1.24	1.40
YK-SE	23	6	0	0.565	0.793	ns	2.24*	26.75	2.00**	2.49**

Populations are listed with their sample sizes (n). Population codes are alphanumeric, with the prefix describing the state or province of collection and the suffix describing the specific sample location. MHC statistics include number of alleles (A), number of private alleles (A_P_), observed (H_O_) and expected heterozygosity (H_E_), deviations from HWE listed as a significance value, and neutrality test values including Tajima’s D, Fu’s Fs, Fu and Li’s D* and F*. Significance values: *p < 0.05; **p < 0.01; ***p < 0.001.

**Table 2:** Summary of genetic variation in 9 microsatellite loci by population

Population	n	Microsatellites				
		A_R_	A_PR_	H_O_ ± (SE)	H_E_ ± (SE)	HWE (loci)
AB-728	24	5.29	0	0.657 (0.059)	0.659 (0.043)	ns
AB-OR	23	5.74	0	0.652 (0.058)	0.645 (0.050)	ns
AZ-30	24	4.98	0.10	0.605 (0.069)	0.606 (0.067)	ns
AZ-KF	24	4.42	0.28	0.625 (0.082)	0.586 (0.072)	* (1)
CO-SJ	24	5.32	0.01	0.648 (0.064)	0.652 (0.048)	ns
CO-ST	24	5.51	0.12	0.648 (0.080)	0.617 (0.059)	ns
KS-BD	24	5.31	0	0.616 (0.067)	0.598 (0.064)	ns
MT-RV	24	5.71	0.22	0.648 (0.052)	0.648 (0.049)	ns
ND-SW	24	5.29	0	0.653 (0.063)	0.631 (0.060)	ns
NV-PC	24	5.19	0.03	0.667 (0.043)	0.663 (0.044)	ns
SD-CU	24	5.31	0	0.704 (0.046)	0.657 (0.043)	ns
SK-14	23	4.8	0	0.560 (0.081)	0.587 (0.064)	ns
SO-CS	24	4.69	0	0.620 (0.069)	0.592 (0.062)	ns
TX-AL	23	5.00	0.06	0.589 (0.074)	0.581 (0.074)	ns
WY-SH	24	5.43	0.19	0.611 (0.070)	0.640 (0.055)	* (2)
YK-SE	23	4.19	0.21	0.541 (0.067)	0.572 (0.061)	* (1)

Populations are listed with their sample sizes (n). Population codes are alphanumeric, with the prefix describing the state or province of collection and the suffix describing the specific sample location. Microsatellite statistics include allelic richness (A_R_), private allelic richness (A_PR_), observed (H_O_) and expected (H_E_) heterozygosity ± standard error (SE), and deviations from HWE (using a False Discovery Rate significance threshold value: *p < 0.0056) listed with the corresponding number of loci [HWE (loci)]

**Fig. 1:**
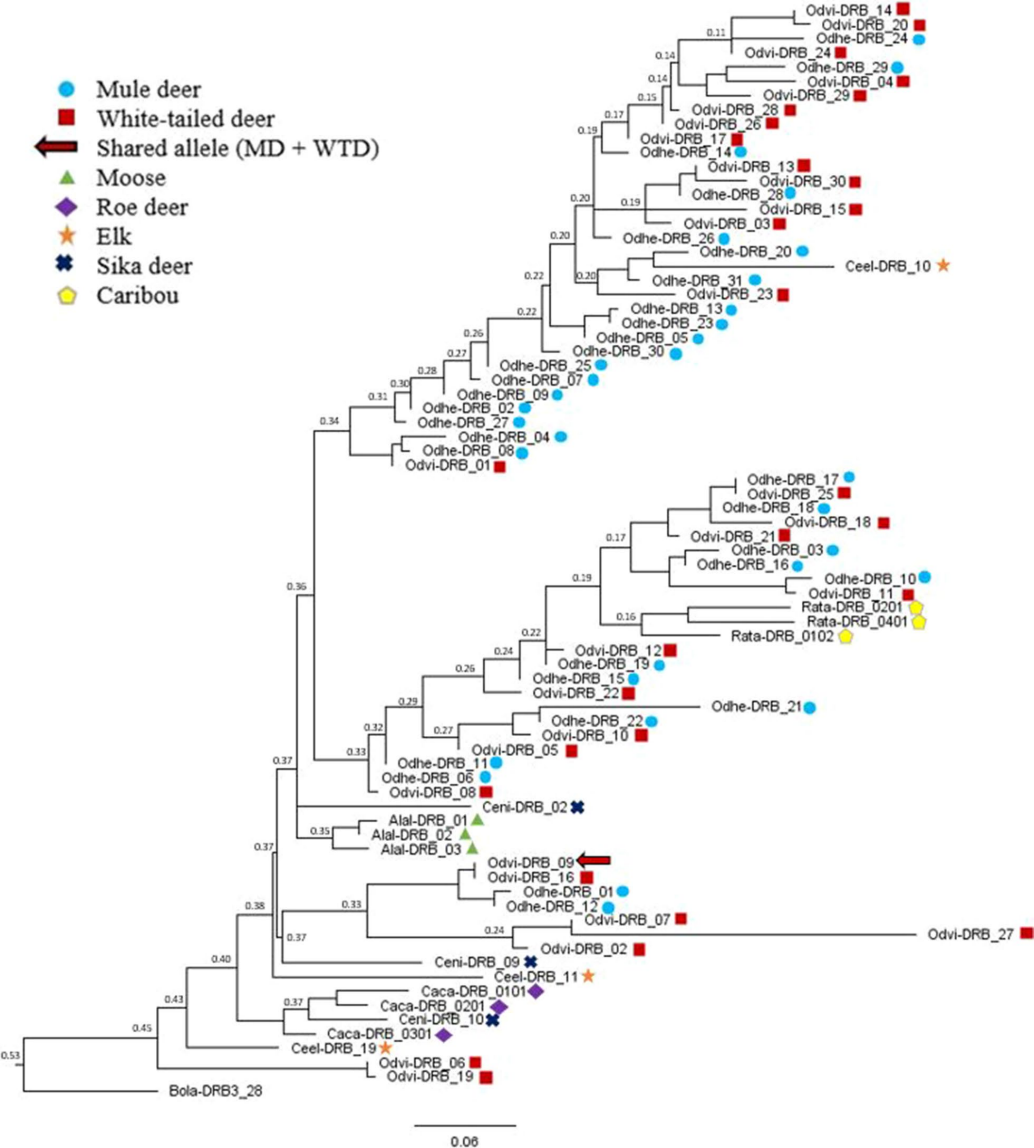
Maximum likelihood phylogenetic tree of MHC DRB exon 2 allele sequences. Sequences included here are the 31 new mule deer alleles (Odhe, *Odocoileus hemionus*) shown by blue circles, 30 white-tailed deer alleles (Odvi, *Odocoileus virginianus*) shown by red squares, and outgroups including moose (Alal, *Alces alces*; green triangles), roe deer (Caca, *Capreolus capreolus*; purple diamonds), elk (Ceel, *Cervus elaphus*; orange stars), sika deer (Ceni, *Cervus nippon*; navy crosses), and caribou (Rata, *Rangier tarandus*; yellow pentagons), rooted with an orthologous cattle DRB sequence (Bola, *Bos taurus*). Nodes are labeled with bootstrap support values. The shared allele between mule deer and white-tailed deer (Odvi-DRB*09) is shown with a red arrow

The maximum likelihood phylogenetic tree showed little evidence for distinct MHC lineages among cervid species (Fig. 1). The bootstrap support values were low (< 0.53) throughout the tree. We observed some clustering of the three moose sequences and a cluster containing all three caribou sequences. Mule deer, white-tailed deer, sika deer, elk, and roe deer were all polyphyletic, though most *Odocoileus* sequences were in a clade separate from other cervids, albeit with weak support (0.34; Fig. 1).

### Population genetic data analysis

**Fig. 2:**
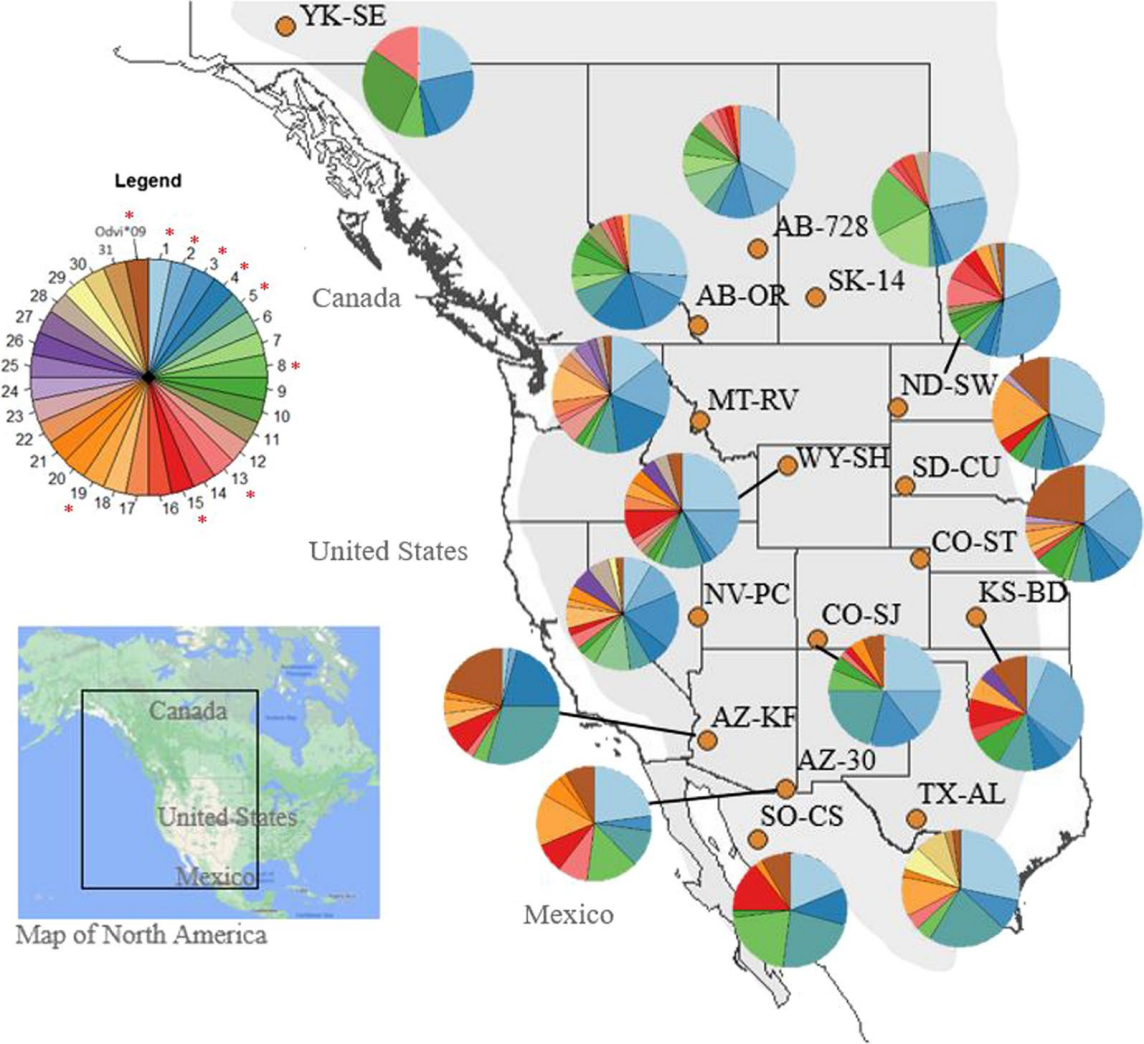
Sampling sites and MHC allele frequencies. 24 individuals were sampled from each of 16 sites spanning the range of mule deer (shown in light grey) between 1995 and 2005. The orange circles show the location of the sampling sites. Site details can be found in Additional file 2. The pie charts represent the proportion of different MHC alleles found in each population, with each allele’s respective color shown in the legend chart (1 = Odhe-DRB*01). The top 10 most common alleles are labeled in the legend with asterisks. Only the most common allele, Odhe-DRB*01, shown in the lightest blue, was found in every population. The shared allele between mule deer and white-tailed deer, Odvi-DRB*09, is shown in the darkest brown

**Fig. 3:**
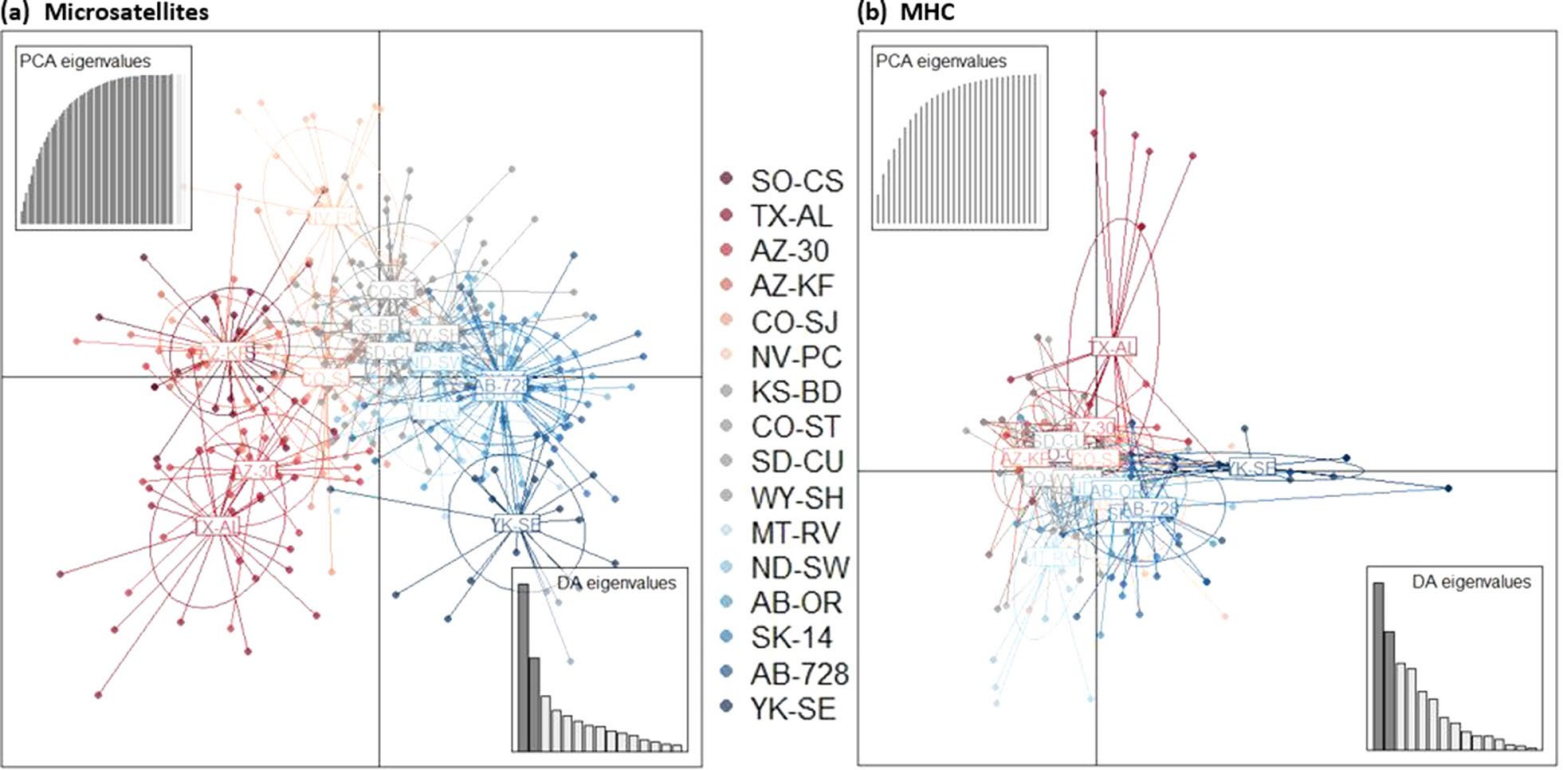
Cluster analysis using Discriminant Analysis of Principal Components (DAPC). Scatterplots show the DAPC of genotypes from the 16 mule deer populations at (**a**) the microsatellite loci and (**b**) the MHC locus. Each individual is represented as a point, colored based on its population of origin, with red being southern or low latitudes and blue being northern or high latitudes. Populations are surrounded by 95% inertia ellipses

MHC allelic richness varied among populations while microsatellite allelic richness remained relatively constant (Tables 1 and 2). The number of observed MHC alleles in each population ranged from 6 in the northernmost population (YK-SE) to 17 at middle latitudes (NV-PC and WY-SH), with only two populations having private alleles (MT-RV and TX-AL; Odhe-DRB*23, Odhe-DRB*25, Odhe-DRB*27, Odhe-DRB*30 and Odhe-DRB*31; Tables 1 and Additional file 1: Table S1). The most common MHC allele (Odhe-DRB*01) was the only allele observed in all populations (Fig. 2, Additional file 1: Table S1). Although white-tailed deer are primarily found in the eastern half of North America, the shared allele between mule deer and white-tailed deer (Odvi-DRB*09) was not restricted to populations along the contact zone (Fig. 2, Additional file 1: Table S1). As with MHC, microsatellite allelic richness was lowest at the most northerly site YK-SE (4.19), but in contrast to MHC diversity, was highest at other northern sites AB-OR and MT-RV (5.74 and 5.71, respectively; Table 2). Private alleles in microsatellite loci were observed in nine of the 16 populations, with the southwestern site AZ-KF having the highest private allelic richness (0.28; Table 2). Overall, allelic richness was positively correlated between MHC and microsatellites (r = 0.697; p = 0.003). Observed heterozygosity ranged more widely for MHC than microsatellites and was not significantly correlated between the two genetic marker types (r = 0.092; p = 0.73). The microsatellite loci were at HWE in most populations (with 4/144 tests deviating from HWE across the 9 microsatellite loci), whereas deviations from HWE were evident at the MHC for 8 of 16 populations (Tables 1 and 2). Neutral and demographic processes are predicted to affect all loci similarly, thus the presence of widespread heterozygote deficiencies relative to Hardy–Weinberg expectations specifically in the MHC suggest selection at that locus. We observed no evidence of genetic structure at either locus type from the STRUCTURE software approach (Additional file 1: Fig. S3). DAPC analysis of both MHC and microsatellites revealed little overall population structure, with most population clusters showing admixture (Fig. 3). We saw a slight latitudinal trend in the microsatellite DAPC scatterplot along axis 1 that was not present in the MHC data (Fig. 3). TX-AL and YK-SE populations were slightly more distinct from other populations in both analyses (Fig. 3).

**Fig. 4:**
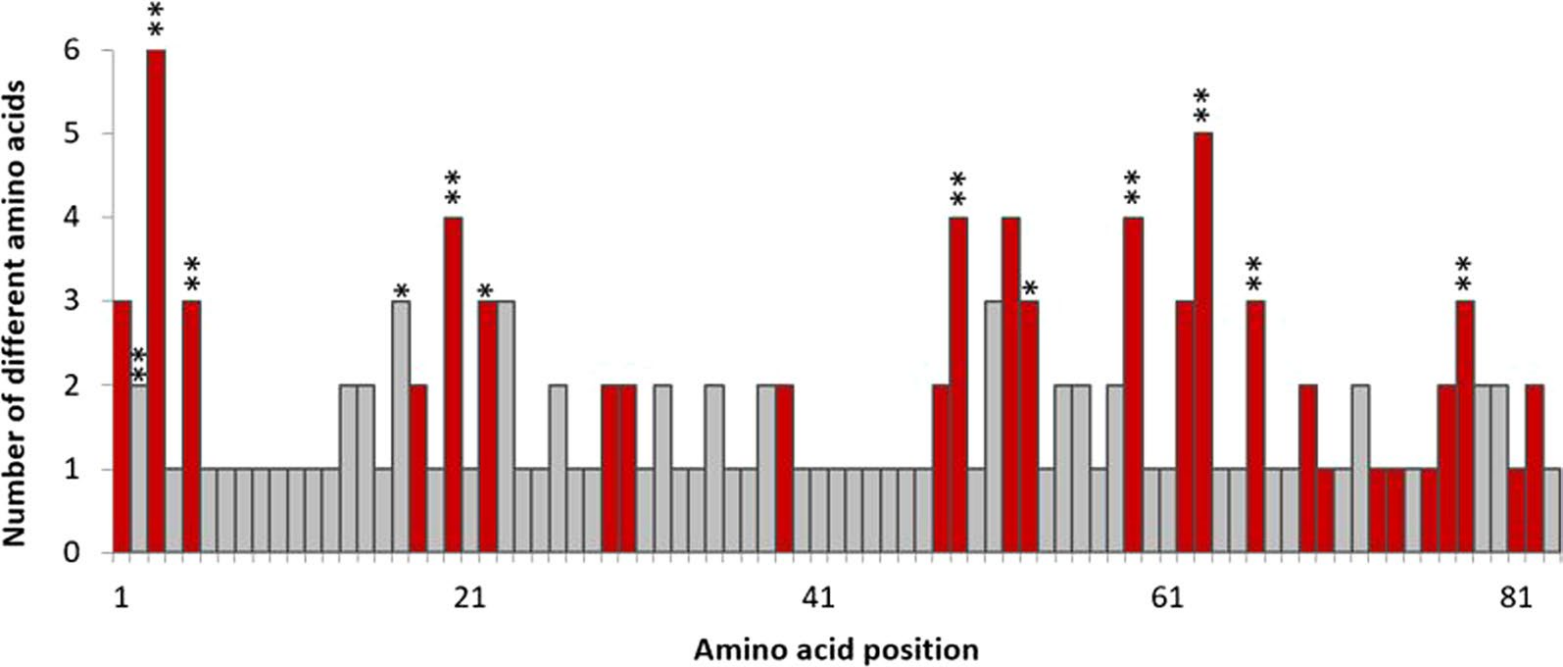
Variation in amino acids for MHC class II DRB exon 2. Amino acid sites corresponding to human HLA-DRB antigen binding sites are shown in red. Positively selected sites using PAML model M8 are denoted by asterisks (*p < 0.05 and **p < 0.01)

We observed evidence for selection on the MHC class II DRB exon 2 in mule deer. dN/dS ratios (ω) above 1 for the full exon (ω = 1.18) and the ABS sites (ω = 2.05) indicate positive selection, whereas non-ABS sites showed evidence of purifying selection (ω = 0.59). Further support for positive selection comes from comparing codon substitution models; models M2a and M8 provided a better fit to the MHC sequence data than M1a and M7 (p < 0.01). Twelve sites were identified as under positive selection in model M8 (posterior probability > 0.95), nine of which were also identified in model M2a (Fig. 4). Ten of the twelve sites identified as being under positive selection corresponded to human HLA antigen binding sites (Fig. 4). Neutrality tests showed positive values across all populations (Table 1). Fu and Li’s D* and F* tests showed six and five populations with statistically significant values, respectively (Fu and Li’s D*: AZ-30, CO-SJ, KS-BD, NV-PC, SK-14, and YK-SE; Fu and Li’s F* significant values included the same as D* except for NV-PC). The YK-SE population showed statistically significant values for Tajima’s D as well as Fu and Li’s D* and F* and the population showed the highest value for Fu’s Fs. Significant values suggest that selection may be driving MHC variation in these populations, and positive values point to balancing selection, recent population bottlenecks, or the presence of intermediate frequency alleles.

**Fig. 5:**
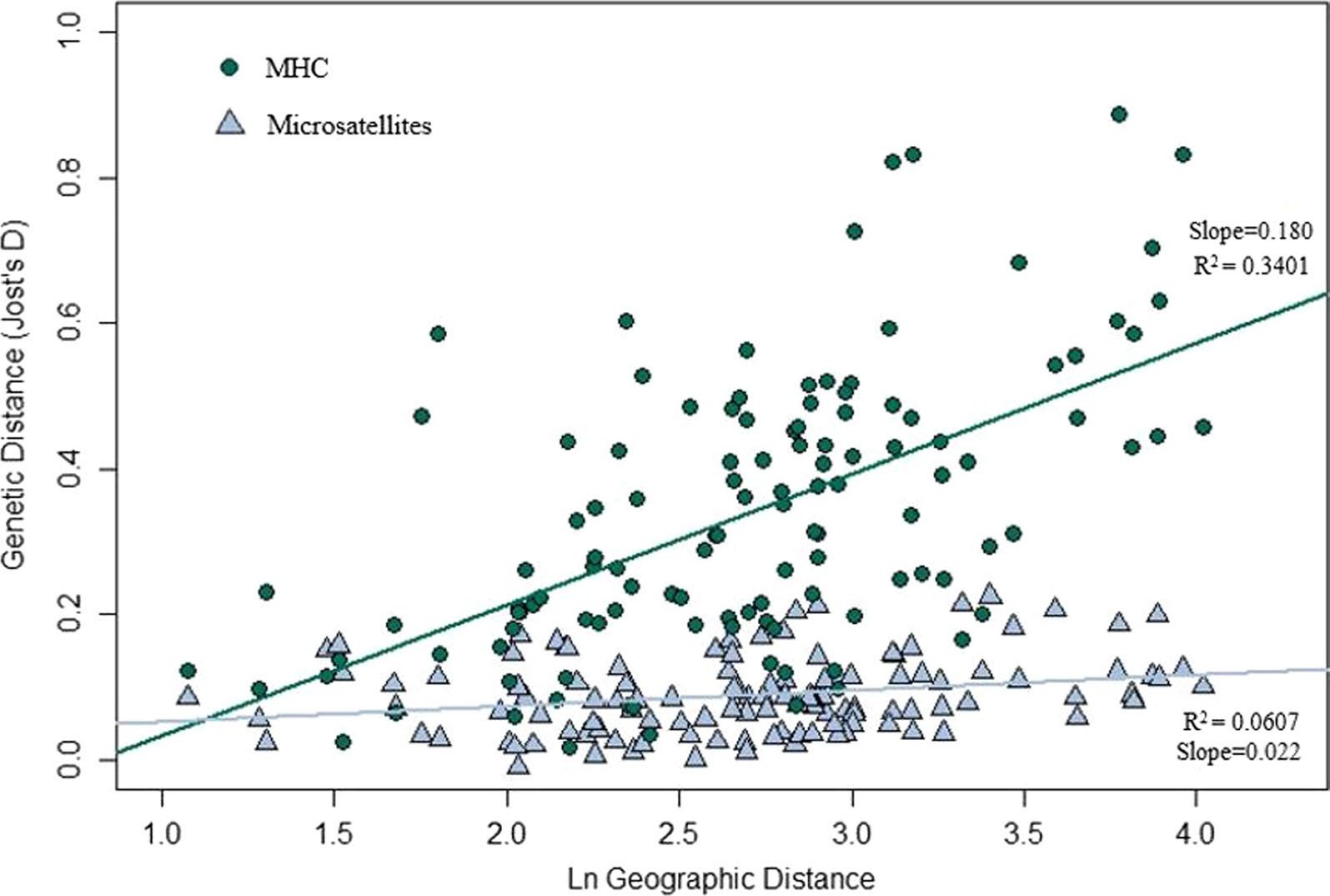
Isolation by distance. The genetic distance (Jost’s *D*) between each pair of populations was calculated for microsatellites (blue triangles) and MHC (green circles) and compared to geographic distance

Genetic differentiation between populations was not driven by the same evolutionary processes in both marker types (Mantel r = 0.10, p = 0.28). Whereas both MHC and microsatellite differentiation could be explained using a simple IBD pattern of gene flow, the slope was significantly steeper for MHC (0.180) than microsatellites (0.022) (p < 0.0001; Fig. 5). Both marker types experience identical gene dispersal patterns, thus stronger IBD (steeper slope) suggests the presence of evolutionary factors that act exclusively on MHC variation.

### Environmental analysis

**Fig. 6:**
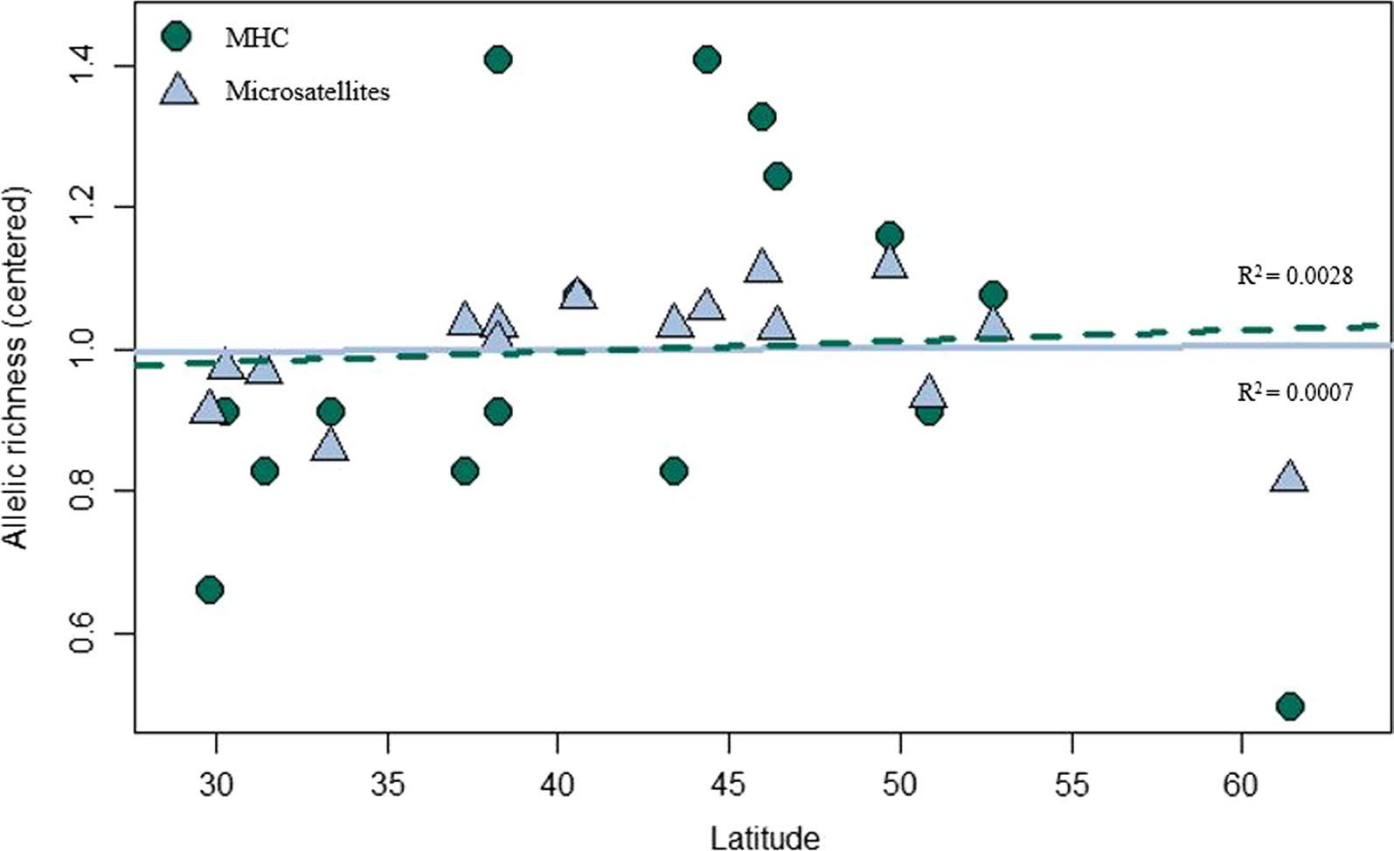
Mean-centered allelic richness and latitude. Mean-centered allelic richness is similar across latitudes for both MHC (green circles, dashed line) and for microsatellites (averaged across loci; blue triangles, solid line)

**Fig. 7:**
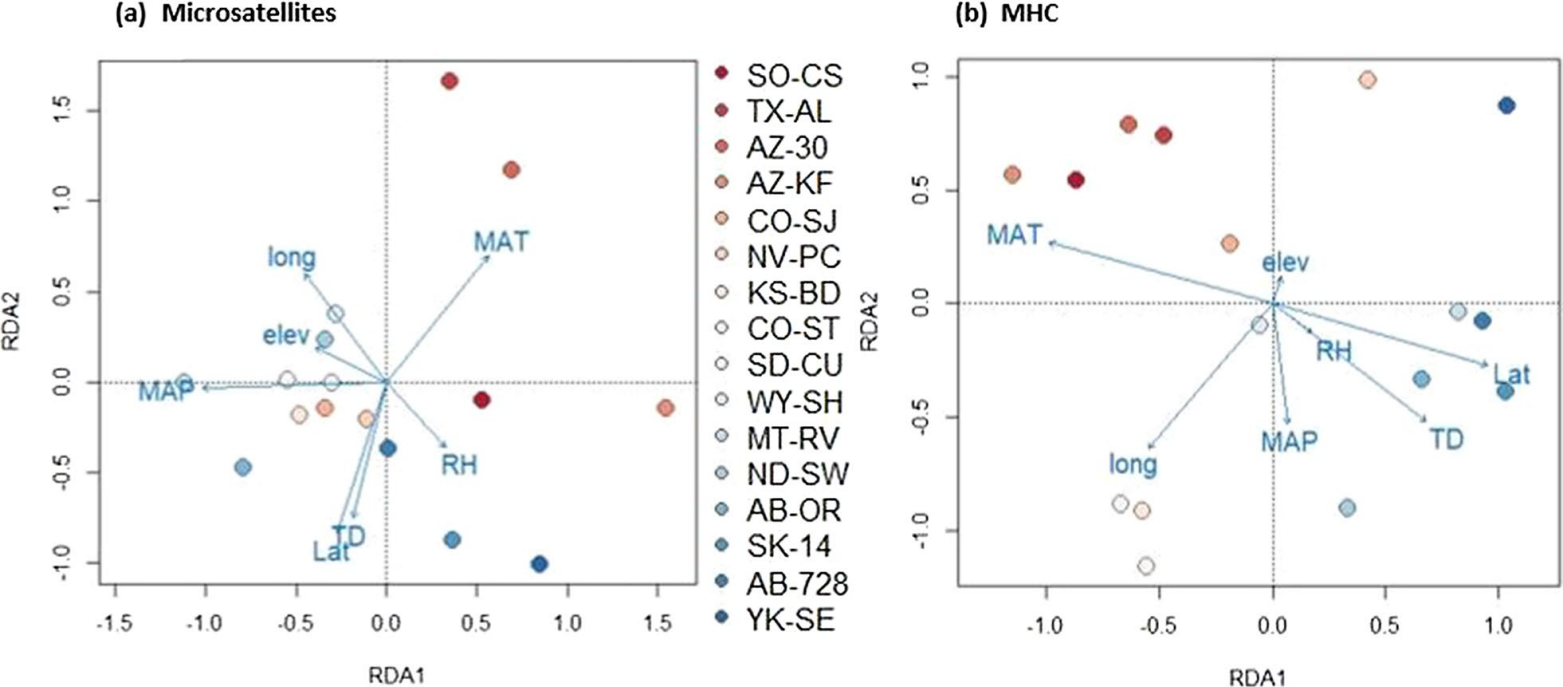
Redundancy Analysis (RDA) for microsatellites and MHC. (**a**) Axes 1 and 2, explaining 46% and 23% of the variance, respectively, in allelic richness for 9 microsatellite loci across 16 populations. (**b**) Axes 1 and 2, explaining 27% and 21% of the variance in allele frequencies for the MHC locus across 16 populations. Points for each plot are colored by population according to latitude. Vectors in blue show constraining axes, or environmental predictors, pointing in the direction of positive contribution, including latitude (Lat), longitude (long), elevation (elev), mean annual temperature (MAT), mean annual precipitation (MAP), temperature difference between warmest and coldest months (TD), and relative humidity (RH)

Although environmental variables may indirectly affect genetic variation through their influence on pathogen abundance and diversity, we found that neither MHC nor microsatellite diversity were significantly correlated with latitude, elevation, or any of the bioclimatic variables we tested. Mean-centered allelic richness values were similar across latitudes (Fig. 6) and a low coefficient of determination (R^2^ = 0.0028 for MHC and 0.0007 for microsatellites) suggests that latitude is not an important driver of among-population variation in allelic richness in our system. Investigation of environmental variables revealed many that were correlated with each other; eleven predictors were removed from analysis leaving latitude, longitude, elevation, mean annual temperature (MAT), seasonality (TD), mean annual precipitation (MAP), and relative humidity (RH) variables in the RDAs. The first two axes in the microsatellite RDA explained 46% and 23% of the variance in allelic richness across populations and both the full RDA model and axis 1 were significant (p = 0.05 and 0.03; Fig. 7a). Populations tended to follow logical patterns in terms of relation to environmental predictors; populations located in southwestern North America tended to be positively related to mean annual temperature and negatively related to mean annual precipitation and seasonality, while northern populations showed the opposite relationships (Fig. 7a). The MHC-based RDA showed similar spatial patterns of populations; sites which had higher latitudes also were negatively related to mean annual temperature, but positively related to seasonality (Fig. 7b). Axes 1 and 2 explained 27% and 21% of the variance, respectively, in allele frequencies for MHC across the 16 populations (Fig. 7b). However, the full RDA model and all axes within the MHC RDA were not significant (all p > 0.1). These results do not provide evidence to support the hypothesis that specific climatic conditions are influencing pathogen load in a way that generates a strong selective force on MHC diversity in mule deer populations.

## Discussion

Understanding the relative effects of natural selection and demography at functionally important immune genes in wildlife is increasingly important as emerging epizootic and zoonotic diseases threaten animal and human health [67, 68]. Our comparative analysis of range-wide variation at the MHC and neutral microsatellite loci in a widespread and mobile species adds much to the developing picture of how evolutionary mechanisms work collectively to shape allelic variation at a broad scale. In this study, we found that while selection plays an inherent role in the evolution of the MHC in mule deer, demography is also a significant predictor of broad scale patterns of MHC variation. Population differentiation was stronger at the MHC than at neutral microsatellite loci, suggesting that there has been selection for locally adapted MHC variation driving differentiation beyond that arising from neutral evolutionary processes (i.e., drift and gene flow). Yet, similar patterns of variation between MHC and neutral loci show that the microsatellite-based signatures of historical demography persist in the face of selection, and that demography is a key contributor to functional diversity at a broad scale, even in large and mobile populations where drift is expected to be weak.

Patterns of polymorphism in the mule deer MHC revealed classic signatures of natural selection. We found high dN/dS ratios and positive neutrality test values in the MHC sequences, on the entire exon and in peptide-binding regions. Tests of codon substitution models also revealed that the codon sites that were under selection closely matched the inferred antigen-binding sites from the human HLA molecule. Our results showing that selection acts on functional codons within the MHC are consistent with previous studies in ungulates and other vertebrates, namely white-tailed deer [69, 70], musk deer [43], red deer [71, 72], moose [58], cattle and other ruminants [21, 73], Nile tilapia [74] and prairie chickens [75].

Signatures of selection that we observed at the MHC were not present in the neutral microsatellite loci. Heterozygote deficiencies at the MHC locus were found in 50% of the mule deer populations surveyed, whereas microsatellites were consistently in Hardy–Weinberg equilibrium (6 loci exhibited no deviations, and the other 3 loci exhibited heterozygote deficiencies in 1 or 2 populations). Other cervids also exhibit heterozygote deficiencies at the MHC DRB locus, including in white-tailed deer, caribou, and moose [58, 66, 69, 76]. An observed heterozygosity excess would be expected if there were a fitness advantage for MHC heterozygotes, through either pathogen-mediated natural selection or sexual selection (MHC-disassortative mating). However, there are several potential reasons why we might see widespread heterozygote deficiencies in our study, including PCR error (null alleles), cryptic substructure (Wahlund effect), or selection against heterozygotes. Our PCR amplification, based on read numbers, shows no evidence for poorly bound primers or errors in sequencing. Cryptic population structure is likely present in mule deer at a fine scale (e.g., family groups), but is likely washed out by range-wide patterns that show a lack of broad-scale population structure [65]. Selection against heterozygotes is rare, but not unheard of. For example, in caribou, high heterozygosity is associated with decreased immunocompetence, offering a potential mechanism limiting excess heterozygosity in ungulates [76]. Further exploration of immunological costs associated with heterozygosity would help determine whether mule deer experience similar pressures.

Another signature of selection in our dataset was stronger MHC divergence over the same geographic space compared to the microsatellites, as seen by the steeper slope in the IBD analysis. This supports the idea that while neutral processes may establish a baseline of genetic diversity across all loci, natural selection drives additional differentiation at the MHC locus. Stronger population differentiation at the MHC than at neutral loci has also been seen in other vertebrates [51–53, 77]. Most previous studies have compared variation in small, isolated, or recently bottlenecked populations [32, 42, 45, 47, 49, 56, 57], where drift can have an outsized impact on adaptive variation [44, 54, 78]. Moose experienced a historical bottleneck that reduced both neutral and MHC variation; low levels of variation are likely maintained by solitary social behavior and low dispersal [58, 79–81]. White-tailed deer also experienced historical bottlenecks, but drift mitigated by accelerated recovery through extensive translocation and the species’ high mobility [82] likely kept MHC diversity high [66, 69]. Mule deer are not known to have experienced severe historical bottlenecks (Latch et al. [83]), and large and well-connected populations would be expected to maintain high variation at the MHC and genome-wide. With negligible genetic drift, differentiation patterns at the MHC are expected to coincide with differences in pathogen prevalence, which are dynamic over both space and time [84]. Examining how MHC variation changes with spatially or temporally varying parasite pressure would yield additional insight into the relative roles of drift and selection (e.g., [24, 85–87]).

Pathogen mediated selection-based spatial genetic structure at the MHC may be mediated by one or more mechanisms including heterozygote advantage, frequency-dependent selection, or varying pathogen pressures. The difference in environmental conditions across populations at a broad scale may foster differences in pathogen abundance and community composition, creating variable selective pressure across the mule deer range [88–90]. The true landscape of parasite-induced selection pressure is not well described in mule deer but in other species has been associated with latitude [86, 91–94], or environmental factors [95], often with latitude serving as a proxy for environmental factors. Spatially heterogenous selection would be expected to maintain polymorphism and facilitate local adaptation [30, 51, 53, 85, 96–98]. High polymorphism across populations at MHC genes may help populations respond to a wide variety of pathogens and increase population fitness, though costs to maintain diversity may impose an upper limit [76]. In mule deer, low rates of gene duplication and allelic richness compared to other mammals suggest that such a trade-off may not be critical in ungulates [18]. Additionally, signatures of local adaptation may be disrupted due to the high rates of gene flow seen in mule deer, which can introduce maladapted genes to a population.

Historical demography works to further shape MHC evolution in mule deer, and its effects are evident even in large and well-connected populations. DAPC analysis revealed similar patterns of genetic structure in both MHC and microsatellite datasets, and neither dataset showed strong correlations with specific environmental variables. Both the microsatellites and the MHC showed patterns of IBD, which suggests that neutral genetic processes are important in shaping diversity at both neutral and functional loci. MHC allelic richness was overall higher than microsatellites, but when corrected for overall levels of variation was similar to microsatellites and did not show statistically significant latitudinal or environmental trends that might be expected if parasite-mediated selection was the driving force behind MHC evolution [93, 99]. The 16 populations studied here all have historically large effective population sizes and are from the same evolutionary lineage, suggesting that they have experienced similar long-term evolutionary histories [65].

There are several reasons why we could see genomic signatures of both selection and demography in this system. One possibility is that high effective population sizes and high levels of standing genetic variation may contribute to natural selection that is widespread and sustained, yet weak. A combination of demographic influence and weak selection across many functional loci has been shown in mule deer, albeit in a different adaptive context [100]. Weak pathogen-mediated selection is feasible in deer, where pathogens are often rare relative to their host or have low virulence [101]. A second possible mechanism where signatures of both selection and demography could be maintained in MHC class II variation is if other genes are important in pathogen response. MHC genes are highly linked in mammals [102, 103], but genomic signatures of selection on different MHC classes have been shown to vary depending on species [104, 105]. Along these lines, a virulent or prevalent pathogen may not trigger a class II response but may be defended against via another pathway, such as toll-like receptors and cytokines that have been associated with disease resistance and survival in wild populations [106–108]. A third possibility is that the MHC diversity we estimated using genomic DNA sequences may not reflect the expressed diversity, thus providing an incomplete picture of selection. For example, a comparison of cDNA and genomic DNA in songbirds revealed fewer expressed MHC-I alleles than predicted by sequence data [109]. Comparing our genomic variation in MHC class II with cDNA diversity would show whether expressed diversity displays stronger signatures of selection in mule deer.

Our phylogenetic analysis of evolutionary relationships between MHC DRB exon 2 alleles in mule deer and other cervids showed MHC alleles from similar species tend to cluster together. The white-tailed deer and mule deer MHC alleles tended to be intermixed but formed their own clade. Our phylogenetic tree was similar to the MHC-DRB2 tree from Ivy-Israel et al. [69], and indeed used many of the same taxa, though our tree had higher bootstrap supports throughout. In both cases, overall support is low throughout the tree, which is expected in the MHC as it is subject to a high degree of homoplasy [110]. Shared alleles across species and polyphyletic grouping appears to be common for the vertebrate MHC [42, 75, 111, 112]. We observed one shared allele between mule deer and white-tailed deer (Odvi-DRB*09). This could be an example of a trans-species polymorphism, where similar historical biogeography between species can lead to shared alleles or similar MHC variation patterns [113, 114]. Because white-tailed deer and mule deer are closely related species that share many pathogens [115], selection may have maintained the shared allele to confer resistance to a common pathogen. Alternatively, it is possible that the shared allele is an independent identical-by-state mutation. The fact that the shared allele is more recently derived according to the phylogenetic tree, and the fact that it is present in populations where white-tailed and mule deer ranges do not overlap suggests that it may not be a true trans-species polymorphism. A broader survey of *Odocoileus* and related species from North America would help determine the extent and evolutionary history of this shared polymorphism. More detailed studies that characterize the pathogen community and connect estimates of fitness for particular alleles at the MHC and other immune genes are needed to better understand how natural selection influences the evolution of the adaptive immune response in ruminants.

## Conclusions

Comparing spatial patterns of variation at functional and neutral loci reveals that both natural selection and historical demography are important mechanisms driving MHC evolution at a broad scale. Our findings for large, well-connected populations add to the emerging picture of how evolutionary mechanisms work collectively to shape functional variation and the evolution of the adaptive immune response.

## Methods

### Sample collection

*Odocoileus hemionus* tissue samples were collected across the species’ latitudinal range, from animals harvested by hunters between 1995 and 2005 (82% of samples collected in 2000–2001). We sampled 24 individuals from each of 16 populations (Fig. 2). Populations were located in the core mule deer range (the ‘MD’ genetic cluster identified in [65]) and outside the known hybrid zone with black-tailed deer in the Pacific Northwest [100, 116]. Sampled animals were at least 1 year old and 63.8% of individuals were male. Sampled animals were unrelated (all pairwise relatedness values across the dataset were < 0.0312).

### Genotyping at microsatellite and MHC loci

DNA was extracted from all 384 samples using a modified ammonium acetate protein precipitation protocol [117]. DNA was assessed by agarose gel electrophoresis and standardized to ~ 10 ng/µL in TLE buffer (10 mm Tris–HCl, pH 8.0, 0.1 mm EDTA). All samples were previously genotyped at a panel of 9 unlinked microsatellite loci (Odh_E, Odh_K, Odh_C, BM848, C273, Odh_G, Odh_P, RT24, T40; [65]), which we used in this study for comparative purposes. Approximately 5% of the tissue samples (n = 20) were genotyped twice to estimate microsatellite genotyping error rate. We identified one instance of allelic dropout in the 200 repeated genotypes (0.5% error rate), in line with previous estimates of error rate for these same loci (0.6%, [118]). The final microsatellite dataset contained 0.4% missing data (15 missing genotypes out of 3840 total genotypes) and exhibited no linkage disequilibrium or evidence for null alleles. We observed no differences in genetic diversity among years (using the only 2 years with sufficient sample sizes) or between sexes, so the total dataset was used for all analyses.

We amplified a 390 bp product containing the entire MHC class II DRB exon 2 in 20 µL Polymerase Chain Reactions (PCRs) containing 10 ng genomic DNA, 0.5 µM each primer (LA31 and LA32; [119]), 200 µM dNTPs, 1 U Phusion DNA polymerase (New England Biolabs), and 1× Phusion buffer. Primers were modified with adapter sequences on both primers and a 10 bp individual barcode on the forward primer. PCRs were performed with an initial denaturation at 98 °C for 30 s, followed by 25 cycles of 98 °C for 15 s, 50 °C for 30 s, and 98 °C for 15 s, and a final extension at 72 °C for 10 min. PCR products were cleaned with a Qiagen PCR Purification Kit followed by Agencourt AMPure XP magnetic beads, and DNA concentration was quantified on a Qubit 2.0 fluorometer. PCR products were pooled into eight pools, each with 48 samples, and bead cleaned twice more. DNA concentration was quantified and checked on an Agilent Bioanalyzer chip for each PCR pool. Sequencing of cleaned PCR pools was performed on a Roche 454 FLX Genome Sequencer using Titanium chemistry at the University of Illinois.

To determine the number of MHC alleles per individual, we used the web server AmpliSAT [120] to de-multiplex samples within pools (AmpliSAS), cluster amplicon sequences within individuals, and filter individual sequences. Because duplication of the MHC-DRB gene is rare in ruminants and has not been seen in *Odocoileinae*, we predicted a single expressed copy of the gene [21, 55, 58, 69, 70, 72, 121]. However, to accommodate possible gene duplication in mule deer, we set the maximum number of alleles per individual to four. Minimum amplicon depth was set to 100 to remove potential artefacts and samples with low reads. We selected the degree of change (DOC) criterion filtering parameter to estimate the number of true alleles based on sequencing depth [122]. All other parameters were set to the default settings. Data from individuals was merged into a single file using AmpliCOMBINE within AmpliSAT. We aligned and translated sequences into amino acids in MEGA7 [123] and no stop codons were observed in any samples.

### MHC phylogeny

We constructed a maximum likelihood phylogenetic tree using IQ-TREE (v2.1.2, [124]) to visualize evolutionary relationships between our mule deer MHC alleles and other cervid MHC alleles. We used ModelFinder [125] as implemented within IQ-TREE to determine the best substitution model based on Bayesian Information Criteria. Branch support was assessed using 1000 ultrafast bootstrap replicates (UFBoot2 [126]), using the F81+F+I+G4 model in IQ-TREE. We visualized the phylogeny with FigTree (v1.4.4). The phylogeny contains the 250 bp MHC-DRB exon 2 sequences from the 31 new mule deer alleles generated in this study, the 30 MHC DRB alleles previously described in white-tailed deer (*Odocoileus virginianus*), and MHC DRB sequences from five outgroup species within Cervidae [moose (*Alces alces*, roe deer (*Capreolus capreolus*); elk (red deer, *Cervus elaphus*); sika deer (*Cervus nippon*); and caribou (reindeer, *Rangier tarandus*); Table 3]. The tree was rooted with an orthologous cattle DRB sequence (*Bos taurus*).

**Table 3:** Taxa used in phylogenetic analysis

	Sample name	GenBank ID	References
Mule deer (*Odocoileus hemionus*)	Odhe-DRB*01-31	MZ450871-MZ450901	This study
White-tailed deer (*Odocoileus virginianus*)	Odvi-DRB*01-15	AF082161-AF082175	[70]
	Odvi-DRB*16-18	AF407169-AF407171	[66]
	Odvi-DRB*19-30	MK952679-MK952690	[69]
Moose (*Alces alces*)	Alal-DRB*01	X82398	[58]
	Alal-DRB*02-03	X83278-79	
Roe deer (*Capreolus capreolus*)	Caca-DRB*0101	U90923-U90925	[73]
	Caca-DRB*0201		
	Caca-DRB*0301		
Elk (*Cervus elaphus*)	Ceel-DRB*10-11	U11110-U11111	[72]
	Ceel-DRB*19	U11119	
Sika deer (*Cervus nippon*)	Ceni-DRB*02	AY679485	Direct submission
	Ceni-DRB*09-10	AY679492-AY679493	
Caribou (*Rangier tarandus*)	Rata-DRB*0102	AF012717	[21]
	Rata-DRB*0201	AF012719	
	Rata-DRB*0401	AF012721	
Cattle (*Bos taurus*)	Bola-DRB3*28	M94928	[127]

### Population genetic data analysis

For each of the 16 populations, we calculated measures of genetic diversity separately for MHC and microsatellites. We used GenAlEx 6.5 [128] to estimate observed (H_O_) and expected (H_E_) heterozygosity, total number of alleles (A), and number of private alleles (A_P_), and HP-Rare [129] to calculate allelic richness (A_R_) and private allele richness (A_PR_). We tested for deviation from Hardy–Weinberg Equilibrium (HWE) in GenAlEx, employing a false discovery rate correction for multiple tests [130]. To explore the genetic structure and connectivity of populations at both locus types, we used STRUCTURE software on the microsatellite and the MHC datasets. We ran five iterations at each K for K = 1 to K = 16 with 50,000 burn-ins and 500,000 iterations, and correlated allele frequencies. We then used individual genotypes in an unsupervised Discriminant Analysis of Principal Components (DAPC [131]), using the adegenet package v2.1.0 in R [132, 133]. We also calculated genetic differentiation (Jost’s *D*, [134]) between pairs of populations using GenAlEx.

We used several selection and neutrality tests to investigate the influence of natural selection on MHC variation in our mule deer samples. We calculated relative frequencies of nonsynonymous (dN) and synonymous (dS) substitutions for dN/dS (ω) ratios using *codeml* in PAML 4.9j [135] which follows the Nei and Gojobori [136] method. Ratios near 1 indicate selective neutrality whereas values of ω > 1 and ω < 1 suggest positive selection and purifying selection, respectively. We calculated ω values for the entire MHC-DRB exon 2, antigen-binding sites (ABS, inferred from human HLA-DRB [137]), and non-ABS. We tested for site-specific positive selection by comparing two pairs of codon substitution models: model M1a (nearly neutral) to M2a (positive selection) and model M7 (beta) to M8 (beta plus ω) [138]. Pairwise likelihood ratio tests were used to identify codons under selection and the Bayes Empirical Bayes (BEB) method was used to calculate the posterior probability and determine significance of the identified codons [139]. Posterior probabilities of > 0.95 were considered supported, and codons were considered to be under positive selection if they were identified by either models M2a or M8. We also performed neutrality tests in each population, including Tajima’s D [140], Fu’s F, Fu and Li’s F* and Fu and Li’s D* using DnaSP v6.12 [141] to further investigate possible selection at the MHC locus.

To compare patterns of diversity between the putatively adaptive MHC locus and the neutral microsatellite loci, we tested for correlations between MHC- and microsatellite-based genetic diversity (allelic richness and observed heterozygosity) and differentiation (Jost’s *D*) using Pearson’s correlation coefficients (r) and Mantel [142] tests with 999 permutations in R. We tested for isolation-by-distance (IBD) in MHC and microsatellites by conducting a Mantel test with 999 permutations between a geographic distance matrix and pairwise genetic distance matrices of Jost’s *D*. An absent or weak IBD correlation suggests high gene flow or selection that favors similar alleles across locations, whereas strong correlations suggest population isolation or divergent selection. We compared IBD patterns between markers with a t-test to gain additional insight into the relative importance of connectivity and selection in this system.

### Environmental analysis

To investigate the indirect role of environmental variation on pathogen load and thus MHC evolution, we examined the relationship between population-level genetic diversity and latitude, elevation, and climatic variables for MHC and microsatellites. Under pathogen-mediated selection, a strong relationship would be predicted where pathogen diversity, physiology, growth, and/or infectivity is tightly coupled to environmental variables. To account for variation in rates of polymorphism between marker types (average 12 alleles per locus in MHC and 5 in microsatellites) we used mean-centered allelic richness [54]. We used a linear regression to quantify the relationship between mean-centered allelic richness and latitude. To quantify environmental influences on genetic diversity, we used redundancy analysis (RDA, [143]). RDA is a multivariate ordination method which can help detect genotype-environment associations (GEAs) and loci under selection. We used latitude (Lat), longitude (long), elevation (elev), and 15 bioclimatic variables for each population obtained from the ClimateWNA database (www.climateWNA.com, [144]) from the years 1981 to 2010. The bioclimatic variables included mean annual temperature (MAT), mean warmest month temperature, mean coldest month temperature, temperature difference between warmest month and coldest month (TD, or seasonality), mean annual precipitation (MAP), annual heat-moisture index [(temperature + 10)/(precipitation/1000)], mean annual relative humidity (RH), and mean temperature and precipitation for each season (winter, spring, summer, and fall). We also obtained elevation for each population from www.mapcoordinates.net/en. We tested for multicollinearity between variables using Variance Inflation Factors (VIF) and removed correlated variables (|r|> 0.8). We ran RDA analyses for microsatellites using allelic richness at each locus, and for MHC using allele frequencies per population. We plotted the RDA axes using the vegan package v2.5-7 in R [145] to visualize how environmental predictors explained the genetic variation at each locus. F statistics were used to test the significance of the full RDA models and each constrained axis.

## Supplementary Information

**Additional file 1: Figure S1.** Sequencing depth and allele calls. **Figure S2.** Alignment of MHC amino acid sequences. **Figure S3.** STRUCTURE results. **Table S1.** MHC allele frequencies and sequencing results by population. **Table S2.** Microsatellite allele frequencies by population.

**Additional file 2.** Sample details, microsatellite and MHC genotypes per individual.

## Data Availability

All data generated or analyzed in the current study are included in Additional file 2. Novel sequences for this project have been deposited in GenBank (MZ450871-MZ450901).

## Abbreviations

A: Number of alleles
ABS: Antigen-binding site
Ap: Number of private alleles
Apr: Private allelic richness
Ar: Allelic richness
BEB: Bayes Empirical Bayes
DAPC: Discriminant Analysis of Principal Components
dN: Rate of nonsynonymous substitutions
DNA: Deoxyribonucleic acid
DOC: Degree of change
dS: Rate of synonymous substitutions
elev: Elevation
GEA: Genotype-Environment Associations
He: Expected heterozygosity
HLA: Human Leukocyte Antigen
Ho: Observed heterozygosity
HWE: Hardy–Weinberg Equilibrium
IBD: Isolation by distance
Lat: Latitude
Long: Longitude
MAP: Mean annual precipitation
MAT: Mean annual temperature
MHC: Major histocompatibility complex
PCR: Polymerase Chain Reaction
RDA: Redundancy analysis
RH: Relative humidity
TD: Temperature difference between warmest and coolest months
VIF: Variance Inflation Factors
