# Supplementary material for: Selection and demography drive range-wide patterns of MHC-DRB variation in mule deer
Source: BMC Ecol Evol. 2022 Apr 6;22:42. doi: 10.1186/s12862-022-01998-8 (PMC8988406; doi:10.1186/s12862-022-01998-8)
Supplement: Supplementary file 1 — Additional file 1: Figure S1. Sequencing depth and allele calls. Figure S2. Alignment of MHC amino acid sequences. Figure S3. STRUCTURE results. Table S1. MHC allele frequencies and sequencing results by population. Table S2. Microsatellite allele frequencies by population. [file 12862_2022_1998_MOESM1_ESM.zip › 12862_2022_1998_MOESM1_ESM/Additional Table and Figure legends 2022.3.8.docx]

**Table S1. MHC allele frequencies and sequencing results by population.**

(uploaded as separate file)

**Table S2. Microsatellite allele frequencies by population.**

(uploaded as separate file)

**Figure S1. Sequencing depth and allele calls.** (a) Sequencing depth compared with number of alleles called per individual sample. (b) Sequencing depth distribution; the average amplicon depth per individual after filtering was 1452, shown with the solid line. The minimum sequence depth was set at 100, shown with the dashed line in both plots. Four samples were removed from analysis due to low read numbers (<100).

**Figure S2. Alignment of MHC amino acid sequences.** The 31 mule deer MHC-DRB sequences and the shared white-tailed deer MHC-DRB sequence are aligned using the standard one-letter codes for each amino acid. The alleles are ordered to match the phylogeny, starting with Odhe-DRB*20. Each (-) indicates that the amino acid in that position is identical to Odhe-DRB*20. Each (+) identifies the positions that are inferred antigen binding sites from human HLA-DRB. The sequences are numbered by amino acid position, from 1 to 83.

**Figure S3. STRUCTURE results.** Mean L(K) plots and bar plots for K=2 to K=4 for MHC and microsatellites respectively.
