# Supplementary figures and images for: Selection and demography drive range-wide patterns of MHC-DRB variation in mule deer
Source: BMC Ecol Evol. 2022 Apr 6;22:42. doi: 10.1186/s12862-022-01998-8 (PMC8988406; doi:10.1186/s12862-022-01998-8)

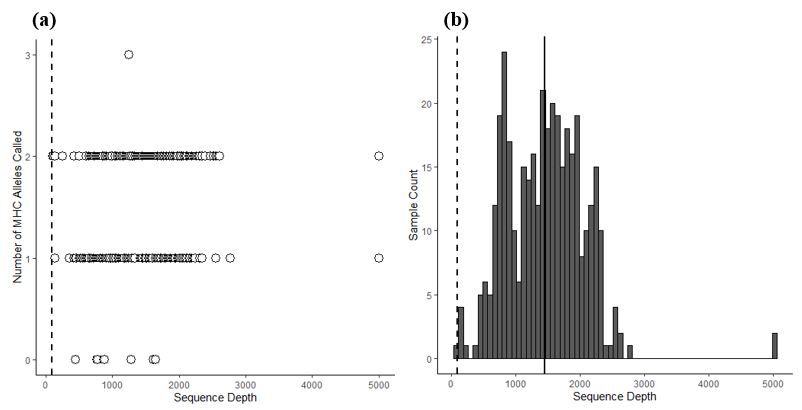

Supplement: Supplementary file 1 — Additional file 1: Figure S1. Sequencing depth and allele calls. Figure S2. Alignment of MHC amino acid sequences. Figure S3. STRUCTURE results. Table S1. MHC allele frequencies and sequencing results by population. Table S2. Microsatellite allele frequencies by population. [file 12862_2022_1998_MOESM1_ESM.zip › 12862_2022_1998_MOESM1_ESM/Figure S1 Sequence depth.JPG]

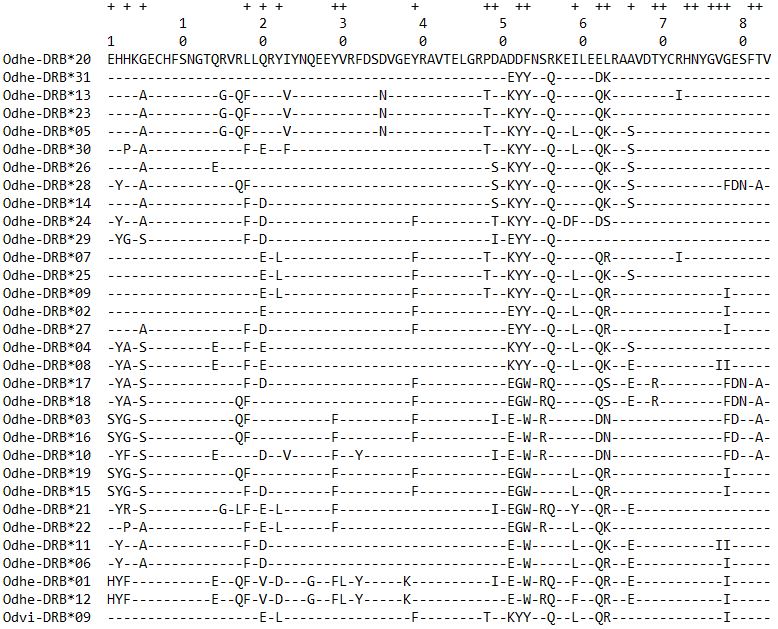

Supplement: Supplementary file 1 — Additional file 1: Figure S1. Sequencing depth and allele calls. Figure S2. Alignment of MHC amino acid sequences. Figure S3. STRUCTURE results. Table S1. MHC allele frequencies and sequencing results by population. Table S2. Microsatellite allele frequencies by population. [file 12862_2022_1998_MOESM1_ESM.zip › 12862_2022_1998_MOESM1_ESM/Figure S2 Alignment of MHC amino acid sequences (phylo order).JPG]

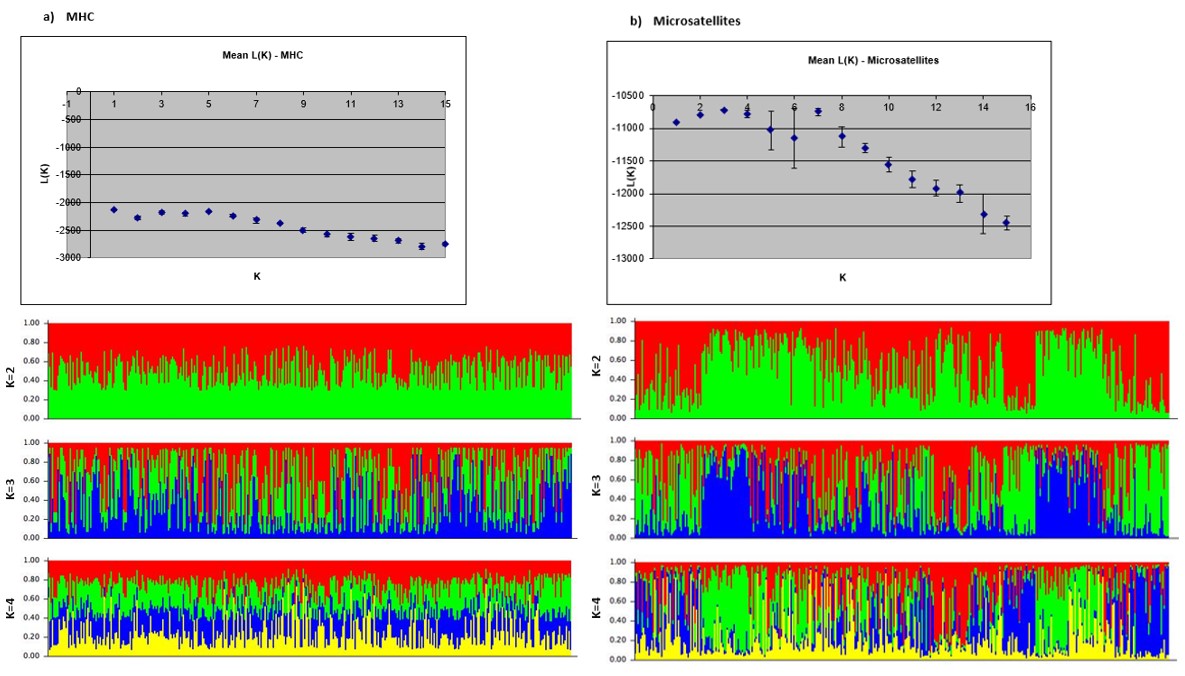

Supplement: Supplementary file 1 — Additional file 1: Figure S1. Sequencing depth and allele calls. Figure S2. Alignment of MHC amino acid sequences. Figure S3. STRUCTURE results. Table S1. MHC allele frequencies and sequencing results by population. Table S2. Microsatellite allele frequencies by population. [file 12862_2022_1998_MOESM1_ESM.zip › 12862_2022_1998_MOESM1_ESM/Figure S3 STRUCTURE results.jpg]
